# Supplementary material for: Polyketide synthase gene clusters in the ericoid mycorrhizal fungus Oidiodendron maius: genome-wide prediction, expression profiling, and functional characterization of knockout mutants
Source: Front Plant Sci. 2026 Sep 16;17:1924430. doi: 10.3389/fpls.2026.1924430 (PMC13623903; doi:10.3389/fpls.2026.1924430)
Supplement: Supplementary file 1 [file SupplementaryFile1.zip › Supplementary Materials/Supplementary Folder 1.zip!/Oidma1_AssemblyScaffolds_Repeatmasked/knownclusterblast/cluster51/jgi.p_Oidma1_35770_mibig_hits.html]

| MIBiG Protein | Description | MIBiG Cluster | MiBiG Product | % ID | % Coverage | BLAST Score | E-value |
| --- | --- | --- | --- | --- | --- | --- | --- |
| ADI24930.1 | VrtE | BGC0000168 | Viridicatumtoxin\_biosynthetic\_gene\_cluster | 60 | 99.2233009709 | 643 | 1.7e-184 |
| EAQ84780.1 | hypothetical\_protein | BGC0001219 | Shanorellin\_biosynthetic\_gene\_cluster | 31 | 38.0582524272 | 120 | 6.5e-27 |
| AGA37280.1 | P450\_monooxygenase | BGC0000819 | Paraherquamide\_biosynthetic\_gene\_cluster | 23 | 91.067961165 | 113 | 8e-25 |
| AIA58897.1 | putative\_cytochrome\_P450 | BGC0001141 | Brefeldin\_biosynthetic\_gene\_cluster | 23 | 93.5922330097 | 86 | 1e-16 |
| AAG27132.1 | Fum6p | BGC0000062 | Fumonisin\_biosynthetic\_gene\_cluster | 29 | 41.5533980583 | 64 | 5.5e-10 |
| ACB12553.1 | Fum6 | BGC0000063 | Fumonisin\_biosynthetic\_gene\_cluster | 29 | 37.8640776699 | 62 | 2.1e-09 |
| NP\_199610.1 | cytochrome\_P450\_705A5 | BGC0000670 | Thalianol\_biosynthetic\_gene\_cluster | 27 | 35.145631068 | 57 | 5.2e-08 |
